# New gain-of-function mutation shows *CACNA1D* as recurrently mutated gene in autism spectrum disorders and epilepsy

**DOI:** 10.1093/hmg/ddx175

**Published:** 2017-05-04

**Authors:** Alexandra Pinggera, Luisa Mackenroth, Andreas Rump, Jens Schallner, Filippo Beleggia, Bernd Wollnik, Jörg Striessnig

**Affiliations:** 1 Department of Pharmacology and Toxicology Center for Molecular Biosciences, University of Innsbruck, 6020 Innsbruck, Austria; 2 Institut für Klinische Genetik; 3 Abteilung Neuropädiatrie, Medizinische Fakultät Carl Gustav Carus, Technische Universität Dresden, 01307 Dresden, Germany; 4 Department I of Internal Medicine, University Hospital of Cologne, 50923 Cologne, Germany; 5 Institute of Human Genetics, University Medical Center Göttingen, 37073 Göttingen, Germany

## Abstract

*CACNA1D* encodes the pore-forming α_1_-subunit of Ca_v_1.3, an L-type voltage-gated Ca^2+^-channel. Despite the recent discovery of two *de novo* missense gain-of-function mutations in Ca_v_1.3 in two individuals with autism spectrum disorder (ASD) and intellectual disability *CACNA1D* has not been considered a prominent ASD-risk gene in large scale genetic analyses, since such studies primarily focus on likely-disruptive genetic variants. Here we report the discovery and characterization of a third *de novo* missense mutation in *CACNA1D* (V401L) in a patient with ASD and epilepsy. For the functional characterization we introduced mutation V401L into two major C-terminal long and short Ca_v_1.3 splice variants, expressed wild-type or mutant channel complexes in tsA-201 cells and performed whole-cell patch-clamp recordings. Mutation V401L, localized within the channel’s activation gate, significantly enhanced current densities, shifted voltage dependence of activation and inactivation to more negative voltages and reduced channel inactivation in both Ca_v_1.3 splice variants. Altogether, these gating changes are expected to result in enhanced Ca^2+^-influx through the channel, thus representing a strong gain-of-function phenotype. Additionally, we also found that mutant channels retained full sensitivity towards the clinically available Ca^2+^ -channel blocker isradipine. Our findings strengthen the evidence for *CACNA1D* as a novel candidate autism risk gene and encourage experimental therapy with available channel-blockers for this mutation. The additional presence of seizures and neurological abnormalities in our patient define a novel phenotype partially overlapping with symptoms in two individuals with PASNA (congenital primary aldosteronism, seizures and neurological abnormalities) caused by similar Ca_v_1.3 gain-of-function mutations.

## Introduction

Autism spectrum disorders (ASD) encompass a number of neurodevelopmental disabilities characterized by restricted and repetitive interests and behavior as well as impaired social interaction and communication. ASD have a worldwide prevalence of about 1% (for review 1), are highly heritable and can be accompanied by cognitive function changes (ranging from above average to intellectual disability), seizures and other comorbidities.

Genomic studies have shown that in addition to common variation, rare genetic variants conferring high disease risk contribute a substantial fraction of ASD risk (2–5). These include chromosomal abnormalities, copy number variations (CNVs) and single-nucleotide variations (SNVs). Next generation sequencing technologies have identified rare *de novo* protein disrupting genetic variants and shown their enrichment in individuals with ASD (2,3,5). Moreover, these studies have used information on rare and recurrent individual *de novo* mutations to pinpoint ASD risk genes (2,3,5). Identification of risk genes also led to the discovery of molecular signaling networks involved in disease pathology, such as chromatin remodeling, synaptic function and transcriptional regulation (2,3,5), providing essential insight into the neurobiology of ASD. In addition, these findings open avenues for novel therapeutic options because understanding of the mutation-induced aberrant signaling defect in a specific patient also enables personalized pharmacotherapeutic approaches. An encouraging example of a successful individualized approach has recently been reported, demonstrating improvement of ASD symptoms and epilepsy in an individual with *TSC2* mutations after treatment with everolimus (6).

We have recently characterized missense mutations in the pore forming α_1_-subunit of Ca_v_1.3 L-type calcium channels (LTCC) (7) identified in two patients with ASD (8,9). Based on the observation that these mutations cause functional changes compatible with a gain-of-function (7), and the known role of these channels for CNS function in rodents (for review 10), we postulated that such gating modifying mutations in *CACNA1D* underlie ASD. The identification of two *de novo* gain-of-function missense mutations in *CACNA1D* in two patients with primary aldosteronism, seizures and neurological abnormalities (PASNA, OMIM: 615474) (11), further points to the involvement of altered Ca_v_1.3 function in neurological disorders. Here, we substantiate these findings by identifying a third *de novo CACNA1D* missense mutation (V401L) in a patient with ASD, intellectual disability and epilepsy which is absent in 60,706 unrelated individuals of the ExAC database (12). In addition to genetic evidence, functional testing revealing pronounced gating changes strongly points to a pathogenic role of this variant. Unlike the vast majority of *de novo* SNVs thought to act with a loss-of-function mechanism, this and other *CACNA1D* mutations lead to gain-of-function, which might be amenable to therapeutic intervention. Here we show that the mutant channel retains full sensitivity to a clinically available LTCC inhibitor (isradipine), suggesting that, like patients with *TSC2* mutations, individuals carrying the V401L mutation may benefit from off-label therapy with a licensed drug. Our findings strengthen the evidence of *CACNA1D* not only as a novel autism risk gene (7) but also as a risk gene for epilepsy (11,13).

## Results

### Clinical report

The patient was the first child of healthy and non-consanguineous German parents. He was born after an uneventful pregnancy in the 42^nd^ gestational week with normal birth weight of 3730 g (44^th^ centile) and occipitofrontal head circumference (OFC) of 36 cm (47^th^ centile). The birth length was 48 cm and thus below normal (−2.25 standard deviation scores).

At the age of 4 months, the child first presented with muscular hypotonia. Profound developmental delay was diagnosed: he could sit at the age of 18 months and he did not learn to walk or talk. He showed autistic behavior and aggressiveness against others and himself. MRI of the brain and the spine at the age of 6 years showed normal brain parenchyma, no microgyria and no malformations. Neurometabolic parameters were normal. At the age of 6 years Autism Diagnostic Interview–Revised (ADI-R) subscores indicated abnormal reciprocal social interaction (score 20), communication (score 14) and repetitive, restrictive and stereotype behavior (score 4). He showed frequent and extensive mannerisms evident as hand flapping and jactations of arms, legs and upper body. Severe intellectual disability was diagnosed. Autism Diagnostic Observation Schedule (ADOS) Module 1 confirmed pronounced autistic symptoms, revealed complete absence of speech and no alternative communication strategies were noted during examination. On examination at the age of 8 years and 1 month, the patient presented with normal measurements (height 131 cm, 54^th^ centile; weight 35.5 kg, 92^nd^ centile; OFC 53.5 cm, 66^th^ centile). He showed no minor anomalies or dysmorphisms, but presented with motoric agitation and stereotypic hand movements. At the age of 2 years he also developed complex focal epilepsy and started showing secondarily generalized seizures at the age of 6 years. Electroencephalograms revealed left hemisphere-predominant multiregional epilepsy-typical abnormalities.

### Identification of a novel *CACNA1D de novo* mutation

To identify the genetic cause of the disorder in the patient, we performed trio sequencing of 4813 known disease genes using the TruSight-ONE kit. No variants in keeping with a recessive mode of inheritance were identified. However, *de novo* analysis identified two variants in the patient which were absent in the parents. The first variant was predicted to cause the c.1430G > A (p.R477H) change in the gene *FASN* (NM_004104). However, this variant is present in dbSNP (rs113931914) and has been identified at low frequency both in the Exome Variant Server and in the ExAC database, indicating that it is likely not the cause of a dominant phenotype. The second variant (chr3:g.53707134G > C, human reference genome hg19) was predicted to cause the c.1201G > C substitution in *CACNA1D* (NM_001128839) and result in a p.Val401Leu (p.V401L) mutation. The mutation was validated with Sanger sequencing and the *de novo* status was confirmed by parental testing. The mutation was predicted to be deleterious by MutationTaster (probability: 0.999998) and Polyphen2 (score: 0.988) and its potential causative role is substantiated by the recent finding that gating-modifying *de novo* mutations have been described in two patients with ASD and intellectual disability as well as in two patients with PASNA (7,11).

### Characterization of the gating properties of *CACNA1D* mutation V401L

**Figure 1:**
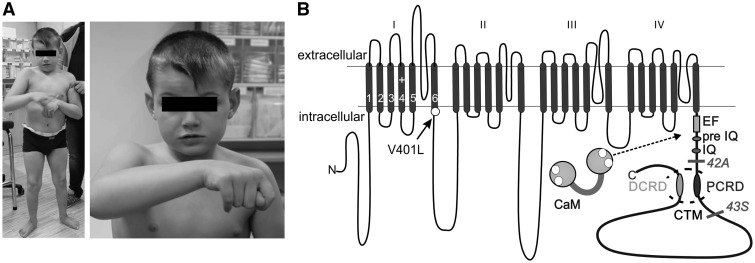
(**A**) The patient carrying a *de novo* missense mutation in Ca_v_1.3 displayed ASD accompanied by developmental delay, epilepsy and muscular hypotonia. (**B**) Transmembrane topology of the pore forming α_1_-subunit. The α_1_-subunit consists of four homologous repeats, each comprising six transmembrane segments connected by extra- and intracellular linkers. Segments one to four of each domain form the voltage sensing domain and segments five to six build the pore (for review see Ref. 48). Mutation V401L (solid arrow), is localized in the activation gate in segment six of domain I in exon 8a, one of two alternatively spliced exons. Furthermore, Ca_v_1.3 undergoes alternative splicing in the C-terminus. Usage of one of two alternative exons, either 42_A_ or 43s results in stop codons (bars) giving rise to two C-terminal short splice variants, partially or completely lacking the C-terminal modulator (CTM) formed by PCRD and DCRD domains (16–18). EF, pre-IQ and IQ are interaction sites for calmodulin (CaM).

The valine mutated in V401L lies in one of two alternatively spliced exons (exon 8a), a highly conserved region within the channel’s activation gate upstream of two previously identified gain-of-function mutations G403D and G407R associated with PASNA and ASD, respectively (7,11). As alterations within the activation gate are known to affect the biophysical properties of Ca_v_1.3 channels (11,14,15), this strongly suggested that mutation V401L might also cause profound gating changes. To test this hypothesis, we determined the properties of Ca_v_1.3 channels carrying the V401L mutation. Since C-terminally long and short Ca_v_1.3 splice variants differing in their gating properties are expressed throughout the brain (16–18), we introduced the mutation in the full-length isoform (WT_L_, V401L_L_) and in the most abundant short variant Ca_v_1.3_43s_ (WT_S_, V401L_S_) (Fig. 1). All mutant α_1_-subunits expressed as a single band with expected molecular mass and without evidence for major changes on protein expression levels as verified by Western Blots (Supplementary Material, Fig. S1, *n =* 8, three independent transfections).

**Figure 2:**
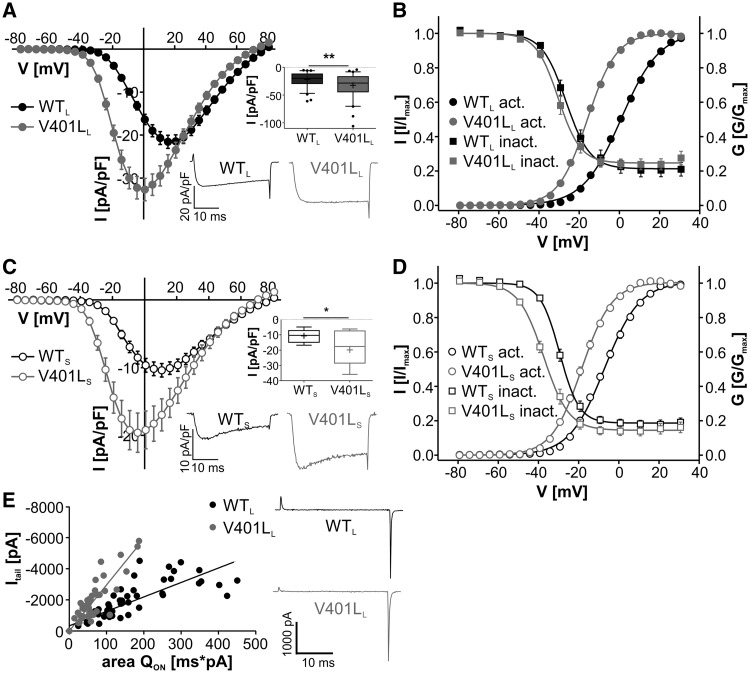
Mutation V401L affects channel gating. (**A + C)** current-voltage relationships (*I_Ca_*, mean ± S.E.M.) of wild-type (WT) vs. mutant C-terminal long (A) and short (C) Ca_v_1.3 splice variants from parallel transfections. Mutation V401L significantly enhanced current densities in both splice variants, also shown by the boxplots and representative traces, obtained from depolarization to the maximal voltage, in the insets (median (5/95 percentile) [pA/pF]: WT_L_: −20.0 (−47.0/−6.21), *n =* 71; V401L_L_: −28.5 (−70.2/−8.18), *n =* 59; *P <* 0.001 against WT_L_; WT_S_: −10.3 (−16.8/−4.86), *n =* 17; V401L_S_: −17.6 (−35.9/−6.16), *n =* 15, *P =* 0.0345 against WT_S_, Mann Whitney test for both). (**B + D**) Voltage dependence of *I_Ca_* activation and inactivation of WT and mutant long (B) and short (D) Ca_v_1.3 splice variants. Mutation V401L significantly shifted voltage dependence of activation and inactivation to more negative potentials in both splice variants. Data shown as mean ± S.E.M., for parameters and statistics see Table 1. (**E**) Ratio of I_tail_ vs. area of ON-gating charge movement (Q_ON_). Mutation V401L_L_ resulted in a higher I_tail_/Q_ON_ ratio, indicating a higher estimated open probability. The following slopes were obtained by linear regression: WT_L_: −9.29 ± 0.82 (R^2^=0.71); V401L_L_: −28.1 ± 1.93 (R^2^=0.8072). Slopes were significantly different (*P <* 0.0001; (**F**) (DFn/DFd)=71.4 (1/104); linear regression, *F*-test, Graphpad PRISM 5.0). Representative traces upon depolarization to the reversal potential are shown to the right. Data were collected from more than three independent transfections. Experiments with poor voltage-clamp were always excluded from analysis.

Mutation V401L resulted in significantly increased current densities and a hyperpolarizing shift of voltage-dependence of activation and inactivation compared to WT in both splice variants (Fig. 2 and Table 1). In the long splice variant Q_ON_ gating charge, as a biophysical measure of channel surface expression, was significantly smaller in the mutant channel (Q_ON_ [pA/pF*ms]: WT_L_: 8.92 ± 0.87, *n =* 43; V401L_L_: 4.71 ± 0.48 *n =* 47; *P <* 0.0001, unpaired student’s *t*-test) despite larger current densities. At a given Q_ON_ maximal tail current amplitude (I_tail_) was larger (Fig. 2E) and mean I_tail_/Q_ON_ ratios were significantly different (I_tail_/Q_ON_ [ms^−1^]: WT_L_: 12.3 ± 0.65, *n =* 43; V401L_L_: 31.9 ± 1.77, *n =* 47; *P <* 0.0001, unpaired student’s *t*-test), an observation compatible with a higher estimated channel open probability (17,19,20). As reported before (16), the estimated open probability and surface expression could not be determined for the short splice variants as the Q_ON_ was too small to be quantified.

**Table 1:** 

Activation					Inactivation			
	V_rev_ [mV]	slope [mV]	V_0.5_ [mV]	*n*	V_0.5_ [mV]	slope [mV]	Remaining current at 30 mV [%]	*n*
*WT_L_*	71.7 ± 0.59	8.94 ± 0.10	0.77 ± 0.54	89	−26.1 ± 0.95	5.86 ± 0.34	21.1 ± 3.78	10
*V401L_L_*	57.8 ± 0.60^a^	6.78 ± 0.09^a^	−15.8 ± 0.57^a^	60	−30.0 ± 1.19^b^	5.20 ± 0.35	25.0 ± 3.45	10
*WT_S_*	71.2 ± 1.08	8.30 ± 0.19	−7.11 ± 0.87	27	−29.1 ± 1.00	4.80 ± 0.43	18.2 ± 2.40	11
*V401L_S_*	60.2 ±1.52^a^	7.15 ± 0.16^a^	−18.5 ± 0.75^a^	22	−37.2 ± 0.78^a^	6.08 ± 0.29^b^	14.4 ± 2.68	10

Data shown as mean ± S.E.M., from > than three independent transfections. Parameters were obtained after fitting normalized (I/I_max_) I-V relationships or normalized (I/I_max_) steady-state inactivation curves, as described in methods. Statistical analysis was performed with unpaired student’s *t*-test, long and short splice variants were analysed separately. V_rev_, reversal potential; V_0.5_, half maximal activation/inactivation; n, n-numbers; WT, wild-type; ^a^*P <* 0.0001, ^b^*P <*0.05, compared to WT respectively.

**Figure 3:**
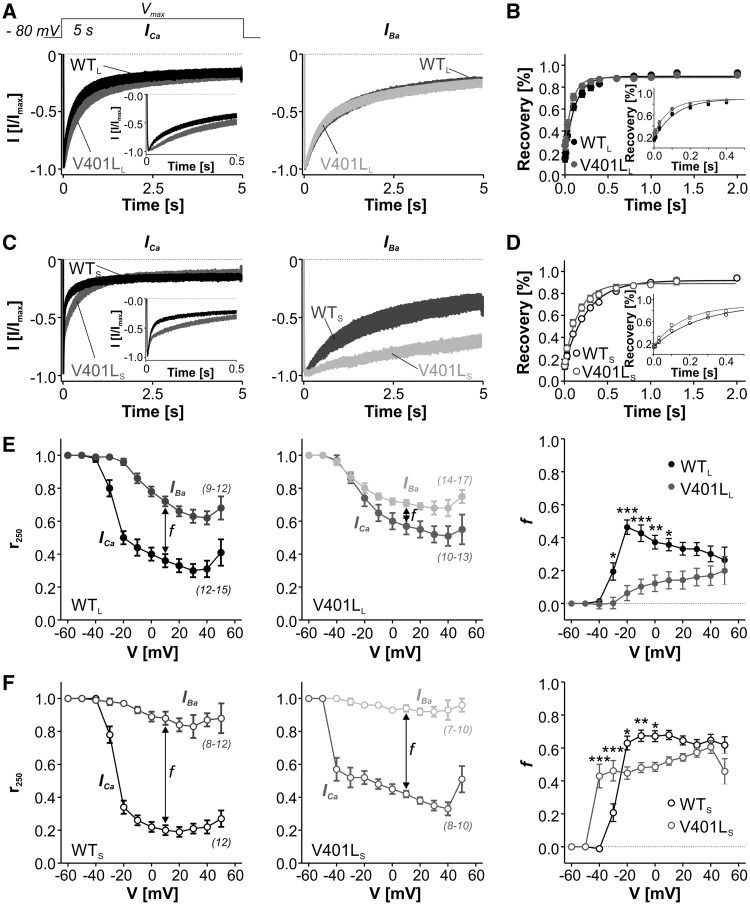
Mutation V401L alters channel inactivation. (**A + B**) 5-s inactivation upon depolarization to V_max_ with Ca^2+^ (left) and Ba^2+^ (right) as charge carrier. Mutation V401L significantly reduced *I_Ca_* inactvation as evident from the inset showing only the first 500 ms, but not *I_Ba_* inactivation when introduced into the long splice variant (A). When present in the short splice, V401L slowed both *I_Ca_* and *I_Ba_* inactivation (B). Traces shown as mean ± S.E.M., for parameters and statistics see Table 1 and 2. (**C + D**) Recovery from *I_Ca_* inactivation (mean ± S.E.M.). Mutation V401L resulted in significantly faster recovery from inactivation in both, long (τ as mean ± S.E.M. [ms]: WT_L_: 121.5 ± 18.3, *n =* 9; V401L_L_: 83.8 ± 6.02, *n =* 11; *P =* 0.048, unpaired student’s *t*-test) (C) and short (τ as mean ± S.E.M. [ms]: WT_S_: 267.2 ± 14.0, *n =* 9; V401L_S_: 167.8 ± 14.3, *n =* 10; *P <* 0.0001, unpaired student’s *t*-test) (D) splice variants. Insets show the first 500 ms. Data were fitted using a mono-exponential equation. (**E + F**) Voltage dependence of Ca^2+^ dependent inactivation (CDI) of WT and mutant long (E) and short (F) splice variants. r250 represents the fraction of the peak current remaining after 250 ms upon depolarization with Ca^2+^ (closed symbols) of Ba^2+^ (open symbols) as charge carrier of WT (left panel) vs. V401L (middle panel), n-numbers are given in parentheses. Parameter *f* represents the difference of r_250_ among *I_Ca_* and *I_Ba_* demonstrating the strenght of CDI (right panel). In both splice variants, mutation V401L resulted in an altered CDI over a broad voltage range, as evident by the *f*-parameters in **E** and **F** (right panel, two way ANOVA of *f*-parameters followed by Bonferroni post-hoc test, *P < 0.05, **P < 0.01, ***P < 0.001, separate analysis for long and short splice variants). Data were collected from more than three independent transfections.

In addition, mutations within the activation gate of Ca_v_1.3 have previously been shown to affect the inactivation of the channel (7,11,14,15). Like other voltage-gated Ca^2+^ channels, Ca_v_1.3 undergoes Ca^2+^- (CDI) and voltage-dependent inactivation (VDI). While VDI is independent from the charge carrier, CDI is almost absent in Ba^2+^ recordings, allowing the separate analysis of both processes. We therefore investigated if mutation V401L altered the inactivation time course of Ca^2+^- (*I_Ca_*) or Ba^2+^ currents (*I_Ba_*) during 5-s long depolarizations to V_max_. As expected, inactivation of *I_Ca_* was faster in WT_S_ in comparison to WT_L_ (due to the absence of CDI inhibition by the C-terminal modulatory domain present only in WT_L_, 16–18) (Fig. 3A and B, Table 2). The mutation slowed *I_Ca_* inactivation in both splice variants (Fig. 3A and B). When introduced into the long splice variant, mutation V401L reduced the fast inactivating component of *I_Ca_* (Fig. 3A), evident by significantly more current remaining after 50 and 100 ms (Table 2). *I_Ba_*-inactivation, however, was unchanged (Fig. 3A, Table 3), indicating that the altered *I_Ca_* inactivation was due to reduced CDI. In contrast, in the short splice variant mutation V401L reduced *I_Ca_* and *I_Ba_* inactivation, revealing a splice-variant dependent effect on VDI (Fig. 3B, Tables 2 and 3). We next analysed CDI over a large range of voltages as the difference of remaining current after 250 ms (r_250_) expressed as *f*-value (Fig. 3E and F). Plotting of *f* against voltage revealed significantly reduced CDI in the mutant V401L_L_ over a broad voltage range (Fig. 3E). When introduced into the short splice variant, V401L resulted in stronger CDI at low (−40 and −30 mV) and weaker CDI at higher potentials (−20 to 0 mV) compared to WT_S_ (Fig. 3F). Like inactivation, also altered recovery from inactivation of Ca_v_1.3 may affect neuronal firing and signaling processes in neurons. Indeed, we found that in both splice variants mutation V401L accelerated recovery of *I_Ca_* from inactivation after 1-s long depolarizations to V_max_ (for statistics see legend to Fig. 3C and D). This was not due to overall reduced inactivation, as differences in *I_Ca_* inactivation among WT and mutant channels only persisted up to 100 ms in response to depolarization to V_max_ (Table 2).

**Table 2:** 

	Remaining *I_Ca_* [%]					
	r_50_	r_100_	r_500_	r_1000_	r_5000_	*n*
*WT_L_*	80.3 ± 3.01	69.3 ± 3.65	37.5 ± 3.97	26.9 ± 3.79	15.9 ± 3.22	14
*V401L_L_*	91.8 ± 2.12^a^	82.7 ± 3.06^a^	46.7 ± 4.67	33.0 ± 4.51	17.5 ± 3.16	15
*WT_S_*	43.7 ± 2.90	35.0 ± 2.84	22.4 ± 2.60	18.6 ± 2.38	14.6 ± 2.17	13
*V401L_S_*	62.0 ± 2.72^b^	54.7 ± 3.01^b^	30.8 ± 3.26	21.2 ± 2.67	11.2 ± 1.73	11

Data shown as mean ± S.E.M., from > than three independent transfections. r values represent the fractions of remaining Ca^2+^ current (*I_Ca_*) after 50, 100, 500, 1000 or 5000 ms upon 5-s depolarization to the voltage of maximal inward current (V_max_). Statistical analysis was performed with unpaired student’s *t*-test, long and short splice variants were analysed separately. n, n-numbers; WT, wild-type; ^a^*P <* 0.01, ^b^*P <*0.001, compared to WT respectively.

**Table 3:** 

	Remaining *I_Ba_* [%]					
	r_50_	r_100_	r_500_	r_1000_	r_5000_	n
*WT_L_*	96.1 ± 1.26	91.1 ± 2.09	63.6 ± 3.60	49.6 ± 3.85	25.5 ± 4.25	10
*V401L_L_*	96.4 ± 0.93	90.5 ± 1.94	62.4 ± 3.35	49.0 ± 3.71	24.9 ± 3.19	12
*WT_S_*	97.2 ± 0.66	95.8 ± 0.88	80.1 ± 3.99	68.0 ± 5.76	35.7 ± 5.57	9
*V401L_S_*	99.3 ± 0.83	98.9 ± 0.83^a^	94.4 ± 1.28^b^	90.5 ± 2.08^b^	70.3 ± 4.01^c^	10

Data shown as mean ± S.E.M., from > than three independent transfections. r values represent the fractions of remaining Ba^2+^ current (*I_Ba_*) after 50, 100, 500, 1000 or 5000 ms upon 5-s depolarization to the voltage of maximal inward current. Statistical analysis was performed with unpaired student’s *t*-test, long and short splice variants were analysed separately. *n*, *n*-numbers; WT, wild-type; ^a^*P <* 0.05, ^b^*P <*0.01, ^c^*P <*0.0001 compared to WT, respectively.

**Figure 4:**
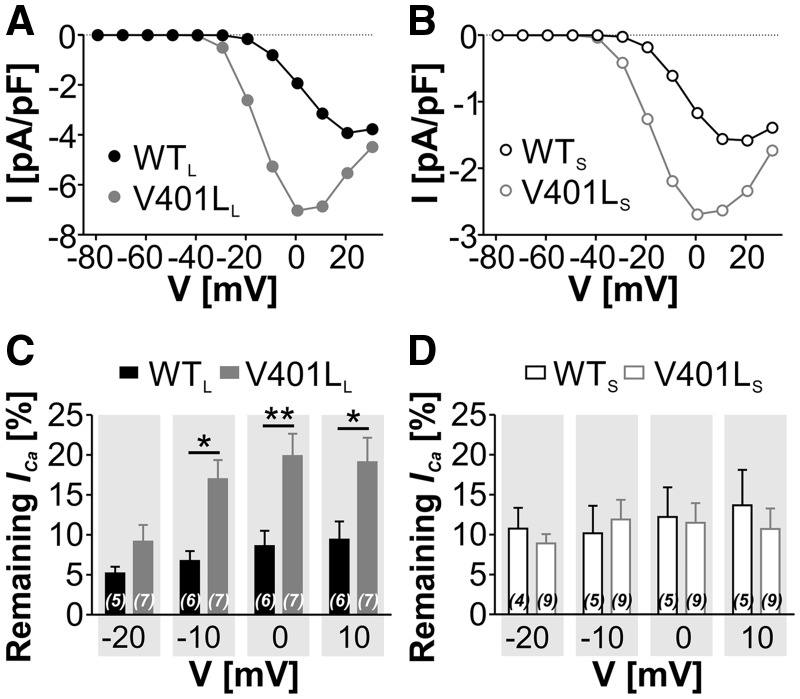
Mutation V401L increases the window current. (**A + B**) Determination of the basal window current for long (A) and short (B) splice variants. Curves were obtained by multiplying the product of the steady-state activation and inactivation in Fig. 2B + D, with the corresponding current densities at the given potentials in Fig. 2A + C, respectively. See Methods for details. Mutation V401L significantly increased the window current in both splice variants (**A**, *P <* 0.05; B, *P <* 0.01; two-way ANOVA). **C + D:** Determination of the persistent current for long (**C**) and short (**D**) splice variants. Remaining current after 5-s depolarizations to different potentials expressed as fraction of maximal possible inward current, assessed by a prepulse to V_max_ in the same sweep. Data shown as mean ± S.E.M., n-numbers are given in parentheses. When introduced into the long splice variant V401L resulted in an enhanced persistent current at − 10 to +10 mV (*P < 0.05, **P < 0.01, two-way ANOVA, followed by Bonferroni post-hoc test).

Since voltage-dependence of activation and inactivation were shifted to more negative potentials this should allow for larger currents elicited by depolarizations from more positive steady-state potentials ("window currents"). As previously described (21), window current at different voltages can be calculated by taking into account the current densities as well as the fractional currents from steady-state activation and inactivation curves (Fig. 2B and D). Mutation V401L resulted in a significantly larger window current at negative voltages in both splice variants (Fig. 4A and B). We also quantified the remaining persistent current after 5-s depolarizations to various potentials and expressed it as the percent of maximal *I_Ca_* measured in a prepulse to V_max_. This predicts changes in persistent current during prolonged depolarizing inputs rather than window current availability upon strong depolarizations as derived from the steady-state inactivation curve above. Mutation V401L increased the fractional persistent current at the end of 5-s depolarizations to −10 to +10 mV but only in the long splice variant (Fig. 4C and D). Taken together, our results clearly demonstrate that the gating changes induced by V401L result in a gain-of-function allowing enhanced Ca^2+^ influx especially at lower membrane potentials. Notice that enhanced window and persistent currents at −30 to +20 mV measured under our experimental conditions with 15 mM Ca^2+^ as charge carrier (Fig. 4) is equivalent to −20 mV more hyperpolarized voltages at physiological extracellular Ca^2+^ concentrations (16) and thus also includes subthreshold membrane potentials in neurons.

### Investigation of dihydropyridine drug sensitivity

Many of the so far identified genetic mutations associated with ASD result in a loss-of-function of the affected gene (3,22). In contrast, V401L and other previously characterized *CACNA1D* mutations identified with ASD present gain-of-function phenotypes (7,23). For patients carrying such mutations clinically available and brain-permeable organic Ca^2+^ channel blockers (e.g. dihydropyridines, DHPs; 24) might thus represent a unique therapeutic option. We therefore investigated if mutation V401L altered the sensitivity towards the DHP isradipine. Since these drugs are strongly voltage-dependent inhibitors (25,26) we compared the response of WT and mutant channels to different concentrations of the DHP isradipine at two holding potentials (−50 and −80 mV). These experiments were only performed on the C-terminal long Ca_v_1.3 splice variant, which is the most abundant one (16) in the brain and shows higher sensitivity towards DHPs than the short isoforms (27).

**Figure 5:**
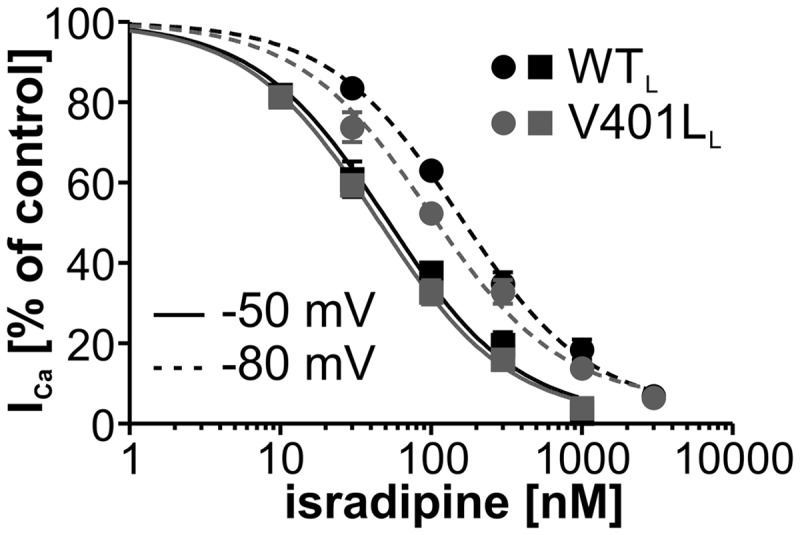
Mutation V401L alters isradipine sensitivity at HP − 80 mV (circles and dashed lines; IC_50_ [%]: WT_L_: 154.7 ± 1.07; V401L_L_: 97.5 ± 1.15; *P =* 0.0278, sum-of-squares F-test) but not at HP − 50 mV (squares and continuous lines; IC_50_ [%]: WT_L_: 51.0 ± 1.17; V401L_L_: 44.5 ± 1.07). Data shown as mean ± S.E.M., fits were performed with Hill slope 1, top was fixed to 1. N-numbers are 8–14 for the individual data points.

At the holding potential of −80 mV, the half maximal inhibitory isradipine concentration (IC_50_) was significantly lower for mutation V401L in comparison to WT but there was no difference in the isradipine sensitivity at −50 mV (Fig. 5, see legend for statistics) suggesting that the mutation increases isradipine affinity for the resting channel state (stabilized at −80 mV). This finding justifies further studies investigating the therapeutic potential of FDA-approved DHPs in the patients with the V401L mutation.

## Discussion

Together with the two previously reported cases (7–9), our discovery of a third patient harboring a Ca_v_1.3 missense mutation, provides strong support for *CACNA1D* as a recurrently mutated risk gene for ASD. Moreover, we strengthen the evidence that *CACNA1D* mutations are also associated with epilepsy (13) and epilepsy with neuromuscular symptoms as described in PASNA patients (7–9,11). In previous studies *CACNA1D* has not emerged as one of the most robust ASD risk genes in large-scale genetic analyses (2,8,28). This is likely due to the fact that genetic studies so far have had a strong emphasis on likely-disruptive genetic variants (including frameshift, nonsense, and mutations in essential spice sites) and heterozygous missense variants causing channel loss-of-function. Haploinsufficiency of *CACNA1D* causes no phenotype in mice and humans (29,30) suggesting that heterozygous loss-of-function variants may confer no or only weak disease risk in humans. This is in contrast to the evidence provided here showing that missense variants inducing gating changes allowing also enhanced Ca_v_1.3 channel activity confer high disease risk for ASD with and without neurological symptoms.

Several observations implicate *CACNA1D* alterations in the pathophysiology of psychiatric and neurological disorders. Our analysis of mutation V401L revealed the characteristic gating changes, allowing enhanced Ca^2+^ entry, as observed in the previously reported germline *CACNA1D* mutations in two patients with ASD (7) and in individuals with PASNA as well as in somatic mutations in aldosterone-producing adenomas (11,15). This supports the pathogenic relevance of such gating changes although it also indicates that they can be part of a disease spectrum including neurological and endocrine (hyperaldosteronism) symptoms.

Strong support for the pathogenicity of such gain-of-function mutations also comes from preclinical data demonstrating Ca_v_1.3 expression in brain areas relevant to ASD as well as a key role for normal CNS function. Ca_v_1.3 is expressed in several brain regions implicated in ASD pathobiology, including the amygdala (31) which together with other areas regulates social behavior (32,33). In addition, Ca_v_1.3 is expressed in the striatum (34,35), linked to stereotypical behavior (36). Ca_v_1.3 channels contribute to neuronal excitability and coupling of Ca^2+^ influx to signaling and gene transcription in neurons (for review 37). At the synaptic level Ca_v_1.3 channels were further shown to play an important role in dendritic spine morphology and refinement (34,38,39), similar to other genes implicated in ASD (for reviews 40,41). Altogether, this strongly suggests that Ca_v_1.3 mutations resulting in altered Ca^2+^ influx can severely affect neuronal function through different mechanisms. Enhanced Ca^2+^ influx through Ca_v_1.3 has recently been shown to result in the formation of filopodia-like dendritic spines in cultured hippocampal neurons (39). Since mutation V401L has the potential to enhance current densities, it may therefore alter dendritic spine morphology which in turn affects the strength and stability of synaptic transmission (for review 41). Moreover, V401L shifted the voltage dependence of activation and inactivation to more negative potentials, which together with the enhanced *I_Ca_* density resulted in an enhanced window current at lower membrane potentials in both splice variants. Given also the faster recovery from inactivation induced by the mutation, the mutant is therefore expected to affect neuronal excitability and signaling. Increased Ca^2+^ inward current could enhance neuronal firing by, for example, promoting plateau potentials (42), but also reduce neuronal excitability through coupling to Ca^2+^ activated K^+^ channels (31). Despite higher window current at lower membrane potentials, the negative shift of the voltage dependence of inactivation in the mutant could also result in a higher fraction of inactivated channels in neurons with a relatively depolarized membrane potential like, for example, dopaminergic neurons of the substantia nigra. Therefore the gating changes described for V401L are predicted to enhance Ca^2+^ channel activity in some and reduce it in other neurons, depending on their activity state. This in turn is expected to result in an excitatory-inhibitory imbalance between neuronal circuits, considered to underlie epilepsy (for review 43).

In contrast to likely-disruptive genetic variants causing loss-of-function, V401L induces gating changes permitting enhanced Ca_v_1.3 activity. This provides us with the unique opportunity to test if inhibition of LTCCs, using brain permeable blockers such as nimodipine or isradipine currently used as antihypertensives (24), could provide therapeutic benefit. We show that mutant V401L retained full sensitivity towards the dihydropyridine Ca^2+^ channel blocker isradipine. This could justify experimental therapeutic attempts with clinically available Ca^2+^ channel blockers in the patient described here as well as the other patients harbouring gain-of-function mutations in *CACNA1D*. An example of such a successful individualized approach has recently been reported, demonstrating improvement of ASD symptoms and epilepsy in an individual with *TSC2* mutations after treatment with everolimus (6).

Another novel finding of our study is that mutation V401L resulted in quantitatively different gating changes and window currents depending on the C-terminal splice variant carrying the mutation. Interestingly, while CDI was reduced in both splice variants, V401L diminished VDI only when introduced into Ca_v_1.3_43s_. Therefore, this mutation may also provide novel insight into splice variant specific voltage-dependent gating mechanisms in further studies.

Mutation V401L further resulted in a reduced area of Q_ON_, indicating a reduced surface expression in tsA-201 cells. Based on our observation that cells expressing V401L channels generally appeared slightly less abundant and were more likely to show a round appearance, we cannot rule out the possibility that increased Ca^2+^ toxicity, as a consequence of enhanced window currents, may select for V401L transfected cells with lower expression levels. However, we also cannot rule out, that mutation V401L to some extent also affects the trafficking of the channel or its stable incorporation into the plasma membrane.

Since Ca_v_1.3 is not only expressed in the brain, missense mutations are also expected to cause other symptoms. However, the patient described here does not show heart, hearing or endocrine dysfunctions, which can in part be explained by the fact that mutation V401L occurs in the alternative exon 8a, which is not expressed in the sinoatrial node or in the inner ear. Based on data from PASNA patients (11) and the discovery of somatic Ca_v_1.3 gain-of-function mutations in aldosterone producing adenomas (15), the patient should instead be closely monitored for hyperaldosteronism.

Taken together, our data strongly support *CACNA1D* as a gene contributing to the risk for a number of psychiatric and neurological manifestations including intellectual disability, epilepsy, neuromuscular symptoms and ASD. Based on these emerging discoveries, we suggest to include *CACNA1D* in genetic panels for diagnoses of neurodevelopmental disorders.

## Materials and Methods

### Genetic testing

The patient was admitted for genetic testing due to profound developmental delay and seizures beginning at the age of two years. Genetic analysis was performed on request of the parents for genetic counselling at the Department for Human Genetics, Technical University of Dresden. Written informed consent for publication of the patient data was obtained from the patient's mother. Array-CGH only revealed one inherited CNV (rsa 11q12.1 (57.532.279−57.899.604)×3pat, 11q12.1 (58.108.903−58.201.583)×3pat) with presumably no functional relevance. Conventional sequencing of the genes *MED12* and *UPF3B* showed no mutation. For further investigation, a total of 50 ng of blood-derived genomic DNA was used to enrich the coding exons of 4813 genes that are associated with known Mendelian disorders. Enrichment was performed using the TruSight-ONE kit (Illumina, San Diego, California, USA) following the manufacturer’s instructions. Subsequently 150 nucleotide paired-end sequencing was carried out on an Illumina MiSeq using v3 chemistry. Reads were mapped to the hg19 human reference sequence. Subsequent variant calling was performed with the CLC Biomedical Genomics Workbench (Qiagen, Hilden, Germany), using the following parameters: minimum coverage = 20; minimum count = 6; minimum frequency (%)=30.0; base quality filter ≥Q20. Target areas were the exons of the 4813 genes plus 10 base pairs of flanking intronic sequence. Variants with a frequency above 1% in the ExAC (Exome Aggregation Consortium) variant database were filtered out. All *de novo* variants, homozygous or compound heterozygous variants as well as inherited or *de novo* X-chromosomal variants were investigated. In parallel, an in-house pipeline based on the Genome Analysis Toolkit was used and the resulting variants were filtered according to their frequency in the general population (<0.01), as reported by dbSNP, the ExAC server, the Exome Variant Server and the 1000 genomes project. Only variants within 10 bp of an exon/intron boundary or predicted to cause a change in the protein sequence were considered. For *de novo* analysis, the alignment files (in bam format) of the parents were compared to that of the child using in-house scripts. Resequencing was performed with Sanger sequencing according to standard protocols.

The new V401L *CACNA1D* variant has been submitted to the Leiden Open Variation Database (LOVD 3.0) as genomic variant #0000163260 (databases.lovd.nl/shared/variants/0000163260).

### Complementary DNA constructs

Human wild-type Ca_v_1.3α_1_-subunits (reference sequence EU363339) comprising alternative exons 8a and 42 (long C-terminal splice variant) or 43s (short C-terminal splice variant, 16) were previously cloned into the pGFP^minus^ vector (no GFP tag, CMV promoter) (16,25). Mutation V401L was introduced into both Ca_v_1.3 splice variants using standard polymerase chain reaction approaches and verified by Sanger sequencing (Eurofins Genomics, Ebersberg, Germany).

### Cell culture and transfection

TsA-201 cells were maintained in culture and transiently transfected with wild-type (WT) or mutant Ca_v_1.3 α_1_- and auxiliary β_3_- (NM_012828) and α_2_δ-1- (NM_001082276) subunits as previously described (44). GFP was co-transfected to visualize transfected cells. Details for Western blot immunodetection of α1-subunits in tsA-201 membrane preparations are described in the Supplemental Information.

### Electrophysiological recordings in Tsa-201 cells

Whole cell patch-clamp experiments have been performed exactly as described previously (7,16,45). Details for recording conditions and data analysis are provided as Supplemental Information.

Window current was determined by multiplying steady-state activation and inactivation to obtain the fraction of available channels at a given potential which was then multiplied with the current density at the respective voltages as described previously (21). Persisting current was quantified as the remaining current after a 5-s depolarizing pulse to different potentials normalized to maximal current measured by a preceding 20 ms pulse to V_max_.

Differences in Ca^2+^ and voltage-dependent inactivation were determined by applying 300 ms long depolarizations to various potentials with either Ca^2+^ or Ba^2+^ as charge carrier. Subsequently, the remaining current at 250 ms was determined and expressed as a fraction of the peak current amplitude as described before (46,47). To quantify CDI, differences of r_250_ values (*f* parameter) of Ca^2+^ versus Ba^2+^ currents at the investigated voltages were assessed.

For pharmacological experiments, cells were perfused by an air pressure-driven perfusion system (BPS-8 Valve Control System, ALA Scientiﬁc Instruments, flow rate: 250 µl/min). Isradipine (a kind gift of Novartis, Basel, Switzerland) was dissolved (10 mM) and serially diluted in dimethylsulfoxide before addition (dilution 1:1000) to the bath solution. Isradipine was applied after at least four constant control sweeps during perfusion with bath solution only. To measure the voltage-dependence of isradipine inhibition (26) cells were depolarized from a holding potential (HP) of −50 mV or −80 mV to V_max_ for 100 ms at 0.1 Hz. Current amplitudes during drug-induced equilibrium were expressed as percent of peak current before drug application. Depending on holding potential and drug concentration drug block was complete after 0.03–2 min. Inhibition experiments were corrected for mean current run-down determined independently for both holding potentials and for all Ca_v_1.3 constructs in separate experiments. Half maximal inhibition (IC_50_) was determined by fitting the data according to the following equation:

y=Bottom+(Top-Bottom)/1+(10  ^  ((X-LogIC50))),

where y is the percent current in the presence of drug and X the log of the drug concentration.

### Statistics

Data were analysed using Clampfit 10.2 (Axon Instruments), Microsoft Excel, SigmaPlot 8 (Systat Software, Inc) and GraphPad Prism 5 software (GraphPad software, Inc). All values are presented as mean ± S.E.M. for the indicated number of experiments (*n*) unless stated otherwise. Data were analysed by unpaired Student’s *t*-test, Mann-Whitney test, two-way ANOVA followed by Bonferroni post-hoc test, *F*-test and one sample *t*-test as indicated in the Figure and Table legends. Statistical significance was set at *P < 0*.05.

## Supplementary Material

Supplementary Data
