# Supplementary material for: New gain-of-function mutation shows CACNA1D as recurrently mutated gene in autism spectrum disorders and epilepsy
Source: Hum Mol Genet. 2017 May 4;26(15):2923–32. doi: 10.1093/hmg/ddx175 (PMC5886262; doi:10.1093/hmg/ddx175)
Supplement: Supplementary Data [file ddx175_supp.zip › Pinggera_2017_Supplemental_Information_HMG_revised_final.docx]

**Supplementary information**

**Supplementary Figure 1**


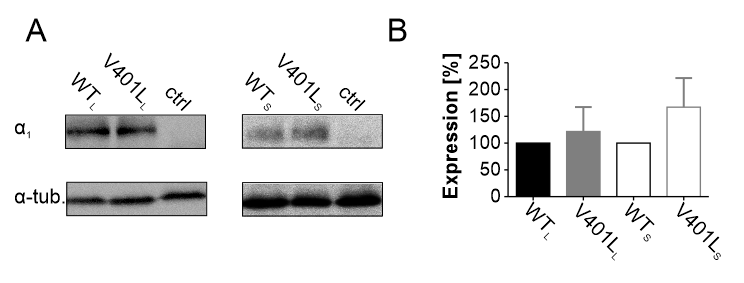


**Figure S1: Expression of Ca_V_1.3 α_1_-subunits in tsA-201 cells.**

**A.** Representative immunoblots from 3 independent transfections performed as described in supplementary materials and methods. For quantification immuno-reactivities of individual α_1_-subunits were normalized to α-tubulin (α-tub.) as loading control. **B.** Quantification of expression levels of mutant Ca_v_1.3 α_1_-subunits normalized to the respective wild-type (WT) (mean±S.E.M. [% of WT]: V401L_L_: 121.9±45.6; V401L_S_: 167.2±54.4; n=8 for each condition, not significant, one sample t-test).

**Supplementary Materials and Methods**

**Electrophysiological recordings in tsA-201 cells**

For whole-cell patch-clamp recordings borosilicate glass electrodes (203-776-0664 Warner Instruments and 64-0792, Harvard Apparatus, USA) with a final resistance of 1.5-3.0 MΩ were pulled using a micropipette puller (Sutter instruments, P-97) and fire polished afterwards (Microforge, Narishinge MF-830). All recordings were performed at room temperature (21-23 °C) in whole-cell configuration using the Axopatch 200B amplifier (Axon instruments), digitized at 50 kHz (Digitizer 1322A, Axon instruments), low-pass filtered at 5 kHz and compensated for 60-90% of the series resistance. The recording solutions contained in mM: *bath*: either 15 BaCl_2_ or 15 CaCl_2_, 10 HEPES, 150 choline-Cl and 1 MgCl_2_, adjusted to pH 7.4 with CsOH; *intracellular*: 135 CsCl, 10 HEPES, 10 Cs-EGTA, 1 MgCl_2_, 4 Na_2_ATP adjusted to pH 7.4 with CsOH. The holding potential (HP) was set to -80 mV. To determine the current-voltage (I-V) relationship, a 30 ms square pulse protocol to different voltages was applied. Resulting I-V curves were fitted to the following equation:

I = G_max_(V-V_rev_)/(1+exp-(V–V_0.5_)/k),

where V_rev_ is the extrapolated reversal potential, V the test potential, I the peak current, G_max_ the maximum conductance, V_0.5_ the half maximal activation voltage and k the slope factor. The voltage-dependence of Ca^2+^ conductance was fitted according to a Boltzman distribution:

G = G_max_/(1+exp(-(V–V_0.5_)/k)).

Estimates for changes in channel open probability and/or single channel conductance were obtained by normalizing the ionic tail current to the amplitude of the integrated ON-gating current (Q_ON_) of the same pulse at the V_rev_. The Q_ON_ at the V_rev_ was also used to determine the surface expression of the channel complexes.

For analysis of current densities, surface expression and estimation of the open probability only recordings from the same transfections were compared.

Steady-state inactivation was measured by applying a control test pulse (20 ms to the voltage of maximal inward current, V_max_) followed by 5-s conditioning steps to various potentials and a subsequent 20-ms test pulse to V_max_ (30-s recovery between protocols). Inactivation was calculated as the ratio between the current amplitudes of the test versus control pulse. Steady-state inactivation parameters were obtained by fitting the data to a modified Boltzmann equation:

G = (1–G_max_)/(1+exp((V-V_0.5_)/k))+G_max_.

Window current was determined by multiplying steady-state activation and inactivation to obtain the fraction of available channels at a given potential which was then multiplied with the current density at the respective voltages as described previously (1). Persisting current was quantified as the remaining current after a 5-s depolarizing pulse to different potentials normalized to maximal current measured by a preceding 20 ms pulse to V_max_.

To investigate inactivation kinetics, cells were depolarized for 5 s to the V_max_, normalized (I/I_max_) and the remaining current (r) at different time points was determined. Recovery from inactivation was determined by a 10 ms test pulses to V_max_ at the indicated time after a 1-s conditioning pulse to V_max_.

Differences in Ca^2+^ and voltage-dependent inactivation were determined by applying 300 ms long depolarizations to various potentials with either Ca^2+^ or Ba^2+^ as charge carrier. Subsequently, the remaining current at 250 ms was determined and expressed as a fraction of the peak current amplitude as described before (2, 3). To quantify CDI, differences of r250 values (*f* parameter) of Ca^2+^ versus Ba^2+^ currents at the investigated voltages were assessed.

For pharmacological experiments, cells were perfused by an air pressure-driven perfusion system (BPS-8 Valve Control System, ALA Scientiﬁc Instruments, flow rate: 250 µl/min). Isradipine was dissolved in DMSO (10 mM), serially diluted in DMSO and diluted 1:1000 into bath solution. Isradipine was applied after at least four constant control sweeps during perfusion with bath solution only. To measure the voltage-dependence of isradipine inhibition (4) cells were depolarized from a HP of -50 mV or -80 mV to V_max_ for 100 ms at 0.1 Hz. Current amplitudes during drug-induced equilibrium were expressed as percent of peak current before drug application. Depending on holding potential and drug concentration drug block was complete after 0.03–2 min. Inhibition experiments were corrected for mean current run-down determined independently for both holding potentials and for all Ca_v_1.3 constructs in separate experiments. Half maximal inhibition (IC_50_) was determined by fitting the data according to the following equation:

y = Bottom + (Top-Bottom)/(1+10^((X-LogIC_50_))),

where y is the percent current in the presence of drug and x the log of the drug concentration.

Leak subtraction was performed either offline (steady-state inactivation, 5-s inactivation, recovery from inactivation and pharmacological experiments) or online using P/4 protocol. Recordings were junction potential corrected by -9.3 mV for Ca^2+^ and by -8.6 for Ba^2+^ as charge carrier, as previously described (5).

**Western Blots and SDS-PAGE**

Western Blots and quantification of total protein expression upon recombinant expression on tsA-201 cells have been performed as described previously (6).

Membrane fractions of tsA-201 cells were prepared as described previously (7). Membrane proteins were denatured in sample buffer under reducing conditions at 57 °C for 15 min. Samples and prestained molecular weight marker (Precision Plus Protein All Blue Standards, Biorad) were separated on polyacryamide gels (5%) and blotted on polyvinylidene fluoride membranes in transfer buffer (25 mM Tris base, 192 mM glycine, 20% (v/v) methanol) containing 0.1% (w/v) SDS. α_1_-subunits were detected using anti Ca_v_1.3 antibody (1:1000, rabbit polyclonal, Alomone labs, CAT: ACC-005, Lot: ACC005AN1902). α-tubulin (anti α-tubulin, 1:100,000, mouse mAb, DM1A, Calbiochem, CAT: CP06, Lot: D00143511) was used as loading control. Peroxidase conjugated goat anti rabbit IgG (1:40,000, Sigma, CAT: A0545, Lot: 54M4812) and goat anti mouse IgG (1:5,000, Pierce, CAT: 31430, Lot: EG767118) were used as secondary antibodies. Immunostained bands were visualized using Pierce ECL Western Blotting Substrate (Thermo Scientific) and a Fusion FX7 Peqlab bioimager. Quantitation of band intensity was performed with Image J 1.46 (National Institute of Health). For quantification integrated density of specific bands was normalized to loading control. Quantification of gel or blot intensities was performed with data obtained within a linear range of exposure. No evidence for proteolytic fragments of α_1_-subunits was found.

**References**

1 Vandael, D.H., Ottaviani, M.M., Legros, C., Lefort, C., Guerineau, N.C., Allio, A., Carabelli, V. and Carbone, E. (2015) Reduced availability of voltage-gated sodium channels by depolarization or blockade by tetrodotoxin boosts burst firing and catecholamine release in mouse chromaffin cells. *J. Physiol.*, **593**, 905-927.

2 Shen, Y., Yu, D., Hiel, H., Liao, P., Yue, D.T., Fuchs, P.A. and Soong, T.W. (2006) Alternative splicing of the Ca_v_1.3 channel IQ domain, a molecular switch for Ca^2+^-dependent inactivation within auditory hair cells. *J. Neurosci.*, **26**, 10690-10699.

3 Liu, X., Yang, P.S., Yang, W. and Yue, D.T. (2010) Enzyme-inhibitor-like tuning of Ca^2+^ channel connectivity with calmodulin. *Nature*, **463**, 968-972.

4 Mahapatra, S., Marcantoni, A., Vandael, D.H., Striessnig, J. and Carbone, E. (2011) Are Ca_v_1.3 pacemaker channels in chromaffin cells? Possible bias from resting cell conditions and DHP blockers usage. *Channels (Austin)*, **5**, 219-224.

5 Lieb, A., Ortner, N. and Striessnig, J. (2014) C-Terminal Modulatory Domain Controls Coupling of Voltage-Sensing to Pore Opening in Ca_v_1.3 L-type Ca^2+^ Channels. *Biophysical journal*, **106**, 1467-1475.

6 Pinggera, A., Lieb, A., Benedetti, B., Lampert, M., Monteleone, S., Liedl, K.R., Tuluc, P. and Striessnig, J. (2015) *CACNA1D* de novo mutations in autism spectrum disorders activate Ca_v_1.3 L-type calcium channels. *Biol. Psychiatry*, **77**, 816-822.

7 Scharinger, A., Eckrich, S., Vandael, D.H., Schonig, K., Koschak, A., Hecker, D., Kaur, G., Lee, A., Sah, A., Bartsch, D. *et al.* (2015) Cell-type-specific tuning of Cav1.3 Ca^2+^-channels by a C-terminal automodulatory domain. *Front Cell Neurosci*, **9**, 309.
