# Supplementary figures and images for: New gain-of-function mutation shows CACNA1D as recurrently mutated gene in autism spectrum disorders and epilepsy
Source: Hum Mol Genet. 2017 May 4;26(15):2923–32. doi: 10.1093/hmg/ddx175 (PMC5886262; doi:10.1093/hmg/ddx175)

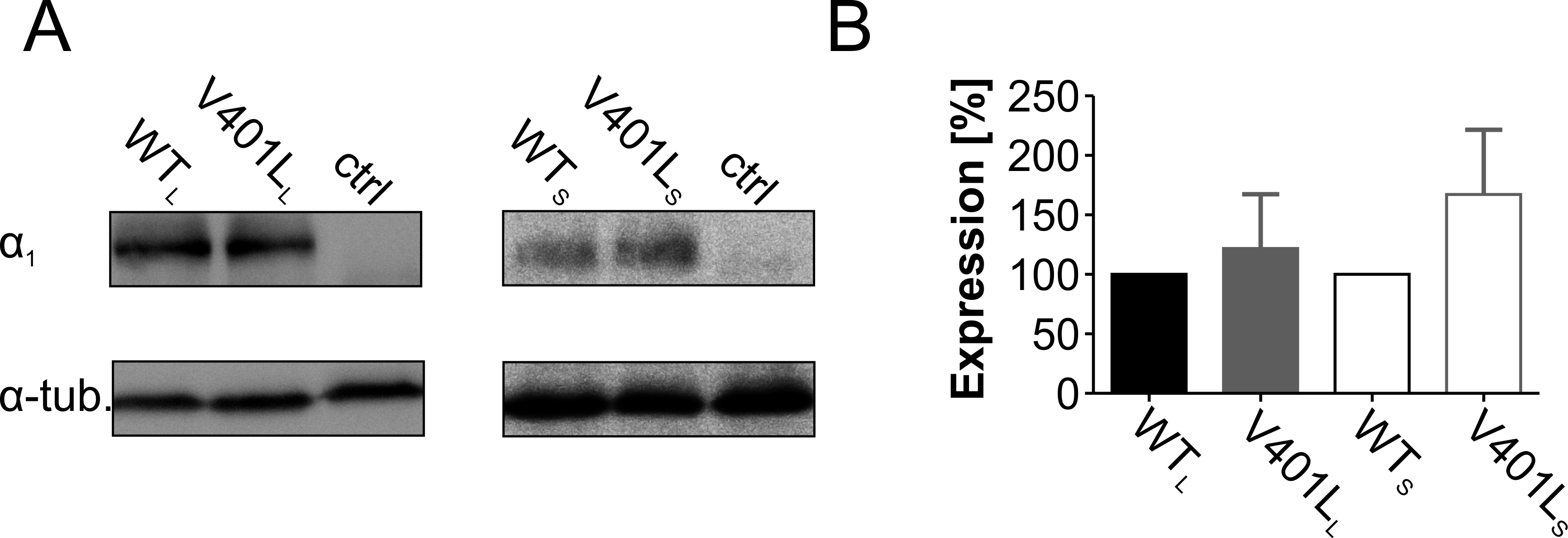

Supplement: Supplementary Data [file ddx175_supp.zip › supplementary figure 1_final.tif]
